# Ceramide activation of RhoA/Rho kinase impairs actin polymerization during aggregated LDL catabolism[S]

**DOI:** 10.1194/jlr.M076398

**Published:** 2017-08-16

**Authors:** Rajesh K. Singh, Abigail S. Haka, Alexandria Brumfield, Inna Grosheva, Priya Bhardwaj, Harvey F. Chin, Yuquan Xiong, Timothy Hla, Frederick R. Maxfield

**Affiliations:** Department of Biochemistry,* Weill Cornell Medical College, New York, NY 10065; Vascular Biology Program,† Boston Children’s Hospital and Department of Surgery, Harvard Medical School, Boston, MA 02115

**Keywords:** atherosclerosis, extracellular hydrolysis, foam cells, lipid and lipoprotein metabolism, macrophages

## Abstract

Macrophages use an extracellular, hydrolytic compartment formed by local actin polymerization to digest aggregated LDL (agLDL). Catabolism of agLDL promotes foam cell formation and creates an environment rich in LDL catabolites, including cholesterol and ceramide. Increased ceramide levels are present in lesional LDL, but the effect of ceramide on macrophage proatherogenic processes remains unknown. Here, we show that macrophages accumulate ceramide in atherosclerotic lesions. Using macrophages from sphingosine kinase 2 KO (SK2KO) mice to mimic ceramide-rich conditions of atherosclerotic lesions, we show that SK2KO macrophages display impaired actin polymerization and foam cell formation in response to contact with agLDL. C16-ceramide treatment impaired wild-type but not SK2KO macrophage actin polymerization, confirming that this effect is due to increased ceramide levels. We demonstrate that knockdown of RhoA or inhibition of Rho kinase restores agLDL-induced actin polymerization in SK2KO macrophages. Activation of RhoA in macrophages was sufficient to impair actin polymerization and foam cell formation in response to agLDL. Finally, we establish that during catabolism, macrophages take up ceramide from agLDL, and inhibition of ceramide generation modulates actin polymerization. These findings highlight a critical regulatory pathway by which ceramide impairs actin polymerization through increased RhoA/Rho kinase signaling and regulates foam cell formation.

A critical initiating event in atherogenesis is the progressive deposition of LDL in the arterial wall (1). This LDL becomes modified, aggregated, and retained. Macrophages encountering such deposits are unable to use standard phagocytic or endocytic mechanisms to catabolize this aggregated LDL (agLDL). Instead, they form an intimate contact with the agLDL, a lysosomal synapse (LS) (2–4). The LS is characterized by local actin polymerization (3), exocytosis of lysosomal contents (2), and regions of low pH at macrophage contact sites with agLDL (2). We have shown previously that actin polymerization is important for formation of the LS, because it drives macrophage plasma membrane contact with agLDL (4). Exocytosis of lysosomal contents into the LS allows delivery of hydrolytic enzymes, such as lysosomal acid lipase and acid SMase residing in the lysosomes, which promote catabolism of agLDL and generation of catabolites such as free cholesterol (3, 4). This free cholesterol promotes macrophage actin polymerization, likely through activation of Rac/Cdc42 GTPases (3). Free cholesterol at the LS that is subsequently internalized by the macrophage promotes foam cell formation (3). We have postulated that release of free cholesterol from macrophages into the extracellular space during agLDL catabolism may be a precursor for the formation of extracellular cholesterol crystals.

Apart from free cholesterol, other metabolites are also produced in the microenvironment of the plaque. In the atherosclerotic plaque, lesional agLDL is known to be rich in ceramide, and it contains 10- to 50-fold-higher content of ceramide when compared with plasma LDL (5). Ceramides are usually found within cellular membranes, and studies have shown that they can act as potent signaling mediators regulating processes such as cell differentiation and proliferation (6). Ceramide can be generated by acid SMase that exists in two forms, a lysosomal acid SMase (L-SMase) that requires a low pH for activity (7) and a secretory acid SMase (S-SMase), which is not localized to the lysosome and can function at neutral pH (8). S-SMase has been reported to be responsible for conversion of sphingomyelin to ceramide in LDL, whereas L-SMase has been implicated in cellular signaling and apoptosis (9). The major pathway of generation of ceramide is through activation of L-SMase, which can cleave sphingomyelin to generate ceramide (10). Generation of ceramide through activation of L-SMase occurs in response to ionizing radiation, UV exposure and TNFα treatment, and can be thought of as a stress signal, inducing cell apoptosis under these conditions (11, 12). Defective function of acid SMase in humans results in Niemann-Pick disease types A and B (13), and interestingly, absence of acid SMase in mice results in the inability to signal apoptosis (14).

Much attention has been paid to the role of ceramide in atherosclerosis, particularly in the vascular system, where it functions as a critical second messenger in many atherosclerotic processes (10). However, few studies have focused on the role of ceramide in macrophage-specific modulation of atherogenic processes. This is surprising because macrophage-derived SMase has been shown to induce partial digestion and aggregation of LDL (15). Furthermore, when ceramide is utilized in studies, a short-chain (C2-ceramide) analog of ceramide is often used because it is more water soluble than are longer-chain, biologically relevant ceramides. In this study, we used both long-chain C16-ceramide (as well as C2-ceramide) and bone marrow-derived macrophages cultured from sphingosine kinase 2 KO (SK2KO) mice, found previously to contain elevated levels of longer-chain ceramides (16). We used these strategies to replicate elevated levels of ceramide that macrophages experience in the microenvironment of the plaque and to investigate the role of ceramide in agLDL catabolism and foam cell formation. We confirm that macrophages accumulate ceramide in atherosclerotic plaques. By taking a microscopy approach, we show that this ceramide can inhibit actin polymerization specifically at the LS and foam cell formation in response to agLDL in a RhoA/Rho kinase-dependent manner. We also show that ceramide from agLDL can be taken up by macrophages and can regulate macrophage agLDL catabolism. These data highlight a novel regulatory pathway that macrophages use to modulate agLDL catabolism and provide a new context in which to view ceramide signaling during foam cell formation and atherogenesis.

## MATERIALS AND METHODS

### Cells and cell culture

J774a.1 macrophages and RAW264.7 macrophages (American Type Culture Collection, Manassas, VA) were maintained in DMEM supplemented with 10% heat-inactivated FBS, 50 U/ml penicillin, and 50 μg/ml streptomycin in a humidified atmosphere (5% CO_2_) at 37°C and used at low passage numbers. Cells were confirmed to be contamination free. Bone marrow-derived macrophages (BMMs) were cultured as follows. Bone marrow was isolated from female mice ages 6–13 weeks of age. We flushed sterilized femurs and tibias from wild-type (C57BL/6), *Sphk1^flox/flox^ Sphk2*^−/−^ (SK2KO), *Sphk1*^−/−^*Sphk2*^−/−^ [Sphingosine kinase 1 (SK1)/2KO], *S1pr1*^−/−^ [Sphingosine-1-phosphate 1 (S1P1) KO], and *S1pr2*^−/−^ (S1P2 KO) mice on a C57BL/6 background [as has been described previously (16–18)] and from *ASM*^−/−^ (A-SMaseKO) and wild-type control [as has been described previously (19)], and cells were differentiated for 7 days by culture in DMEM supplemented with 10% heat-inactivated FBS, 50 U/ml penicillin, and 50 μg/ml streptomycin, supplemented with 20% L-929 cell-conditioned media in a humidified atmosphere (5% CO_2_) at 37°C. Control and KO mice were housed in the same facility, in a pathogen-free environment at Weill Cornell Medical College, and used in accordance with protocols approved by the Institutional Animal Care and Utilization Committees.

### Reagents

AlexaFluor546 (Alexa546), LipidTOX Green, LipidTOX Deep Red, Alexa488-phalloidin, Alexa647-phalloidin, and BODIPY-FL-C5-ceramide were purchased from Invitrogen. C16-ceramide (d18:1/16:0) and C2-ceramide (d18:1/2:0) were purchased from Avanti Polar Lipids, Inc. (Alabaster, AL). Monoclonal anti-ceramide antibody (clone MID 15B4) and desipramine were purchased from Sigma-Aldrich (St. Louis, MO). Y-27632 was purchased from Tocris Bioscience (Bristol, UK). Rho inhibitor I was purchased from Cytoskeleton, Inc. (Denver, CO). Phospho(Thr18/Ser19)-Myosin Light Chain 2 antibody was purchased from Cell Signaling Technology (Danvers, MA). Anti-RhoA and anti-calnexin antibodies were purchased from Abcam (Cambridge, MA). pcDNA3-enhanced green fluorescent protein (EGFP) was a gift from Doug Golenbock (Addene plasmid #13031). pcDNA3-EGFP-RhoAwt [WTRhoA-green fluorescent protein (GFP)] and pcDNA3-EGFP-RhoAQ63L (CARhoA-GFP) were a gift from Gary Bokoch (Addgene plasmid #12965 and #12968).

### Hyperlipidemic ApoE^−/−^ mice

*ApoE*^−/−^ mice were obtained from Jackson Laboratories and placed on a high-fat diet (21% milk fat, 0.15% cholesterol; Harlan Teklad) for 13 weeks. Mice were euthanized and perfused with PBS, and aortas were taken for sectioning.

### Aortic fixation and sectioning

Aortas were fixed overnight in 3% paraformaldehyde at 4°C. Fixed aortas were placed in a solution of 30% sucrose in PBS and stored at 4°C overnight. Aortas were then gently agitated in embedding media (1:2 ratio of 30% sucrose in PBS in OCT medium) and then frozen in the same media using 2-methylbutane and liquid nitrogen. Samples were then cut into 8 μm sections using a Cryostat, mounted onto glass slides and coverslips attached using Vectorshield mounting medium for fluorescence (Vector Laboratories, Burlingame, CA).

### Immunohistochemistry

After blocking with 10% goat serum for 1 h, macrophages were identified using a rabbit polyclonal antibody for F4/80 (Abcam ab100790, Cambridge, MA) at 1:300 dilution overnight at 4°C and AlexaFluor546 anti-rat secondary antibody (Invitrogen) at 1:400 dilution for 2 h at room temperature. Ceramide was stained using a mouse monoclonal antibody (clone MID 15B4) at 1:100 dilution overnight at 4°C and AlexaFluor488 anti-mouse secondary antibody (Invitrogen) at 1:400 dilution for 2 h at room temperature. All antibody labeling was carried out in PBS containing 3% goat serum. Images were acquired with a Zeiss LSM 510 laser-scanning confocal microscope (Thornwood, NY) using a 63 × 1.4 NA objective.

### Lipoproteins

Human LDL was prepared from donor plasma as has been described previously (20). AcLDL was purchased from Alfa Aesar (Haverhill, MA). LDL was labeled using succinimidyl esters of Alexa546. LDL was aggregated by vigorous vortexing for 30 s (21). BODIPY-FL-C5-ceramide was incorporated by incubating 10 μM concentration complexed with BSA (1:1) in 1 mg/ml Alexa546-LDL at room temperature with slow rotation for 1 h prior to aggregation. The pellet was washed once with serum-free DMEM prior to incubation with cells.

### Confocal microscopy

For imaging, cells were plated on poly-D-lysine-coated glass-coverslip-bottom dishes. Images were acquired with a Zeiss LSM510 or LSM880 laser-scanning confocal microscope using a 40× Air, 0.8 NA, or 40× Oil, 1.3 NA, objective respectively. For actin measurements, *z*-stacks were obtained with a step size of 0.98 μm. All data were analyzed with MetaMorph image analysis software (Molecular Devices, Dowingtown, PA).

### Assessment of cell morphology

All measurements were made using MetaMorph software. Cell area was measured by outlining cells. Elongation index was calculated by measuring the maximum cell length and dividing by maximum cell width.

### Immunostaining and foam cell formation

For ceramide immunostaining, BMMs were fixed with 3% (w/v) paraformaldehyde in PBS for 20 min at room temperature, washed extensively with PBS, and subsequently blocked/permeabilized for 1 h at room temperature using 10% goat serum by 0.05% (w/v) saponin in PBS. Cells were then stained with anti-ceramide antibody (1:100 dilution) overnight at 4°C. Cells were washed extensively in PBS prior to staining using Alexa488-anti-mouse secondary antibody (1:400) for 30 min at room temperature. All staining was performed in 3% goat serum by 0.05% (w/v) saponin in PBS. Cells were washed extensively with PBS prior to imaging. For foam cell formation, BMMs were treated for 24 h with Alexa546-agLDL, fixed with 3% (w/v) paraformaldehyde in PBS for 20 min at room temperature, washed with PBS, and then stained using LipidTOX Green or LipidTOX Deep Red (at 1:1,000 dilution in PBS) for 15 min at room temperature followed by extensive washing in PBS prior to imaging.

### Actin polymerization

Cells were left untreated or pretreated where indicated prior to treatment with Alexa546-agLDL for 1 h. Cells were fixed with 3% (w/v) paraformaldehyde in PBS for 20 min at room temperature and stained for filamentous actin (F-actin) using 0.02 U/ml of Alexa488-phalloidin or Alexa647-phalloidin in 0.5% (w/v) saponin in PBS for 1 h. Cells were washed extensively with PBS and then imaged.

### Plasmid propagation and transfection

Plasmids obtained from agar stab cultures were purified using plasmid DNA maxiprep kits (Qiagen) according to manufacturer’s instructions. RAW264.7 macrophages were transiently transfected using Fugene HD reagent (Promega, Madison, WI). Two micrograms plasmid and 6 µl Fugene reagent were added to 100 µl serum-free DMEM, gently mixed and then incubated at room temperature for 15 min. Eight hundred microliters complete DMEM culture media was added to this, mixed gently, and added to macrophages on coverslip dishes. Cells were then cultured for 24 h prior to agLDL treatment.

### SiRNA mediated knockdown of RhoA

WT and SK2KO BMMs were transfected with siRNA using the Amaxa nucleofector I device (Lonza, Basel, Switzerland) using Amaxa Cell Line Nucleofector Kit T (Lonza). Four–5 × 10^6^ cells were resuspended in 100 µl nucleofector solution. Control scrambled all-stars negative siRNA (Qiagen) or a pool of four different siRNA sequences targeting RhoA (Qiagen) were added to a final concentration of 2 µM. Cells were added to cuvettes and nucleofected using program T-20. Cells were transferred to prewarmed culture media and plated into tripartition petri dishes and cultured for 48 h. Cell solutions were trypsinized and plated into coverslip microscopy dishes for microscopy experiments or six-well plates overnight for assessment of knockdown efficiency. Typically, RhoA protein levels 72 h posttransfection were reduced ≥60% by RhoA siRNA in comparison with scrambled siRNA control.

### Image quantification

Actin polymerization was assessed, as has been described previously (3, 4). In brief, *z*-stacks were acquired and a binary mask was created for each slice in the *z*-stack using Alexa546-agLDL signal intensity. This binary mask was applied to the Alexa488-phalloidin image, and the integrated Alexa488-phalloidin fluorescence colocalized with Alexa546-agLDL per *z*-slice was obtained. These values were summed for the entire stack to obtain the total integrated Alexa488-fluoresence colocalized with Alexa546-agLDL per *z*-stack. This was then divided by the number of cells in the field. For assessment of neutral lipid content, images were thresholded to exclude any fluorescence not associated with lipid droplets. Then the integrated LipidTOX Green fluorescence per field was quantified and divided by the number of cells in the field. For quantification of the extracellular agLDL/field, we outlined cells and measured the total integrated fluorescence of agLDL within the cell boundaries, then measured the total integrated fluorescence of agLDL per field and subtracted the intracellular fluorescence from the total fluorescence to obtain extracellular agLDL fluorescence per field. We then expressed this as a percentage of total agLDL per field.

### Statistics

For pairwise comparisons, Student’s two-tailed *t* test was performed. For comparisons of more than two groups, a one-way ANOVA followed by Bonferroni correction was performed. All statistical comparisons were performed using Excel software.

## RESULTS

### Lesional macrophages contact ceramide in an atherosclerotic plaque

**Fig. 1.**
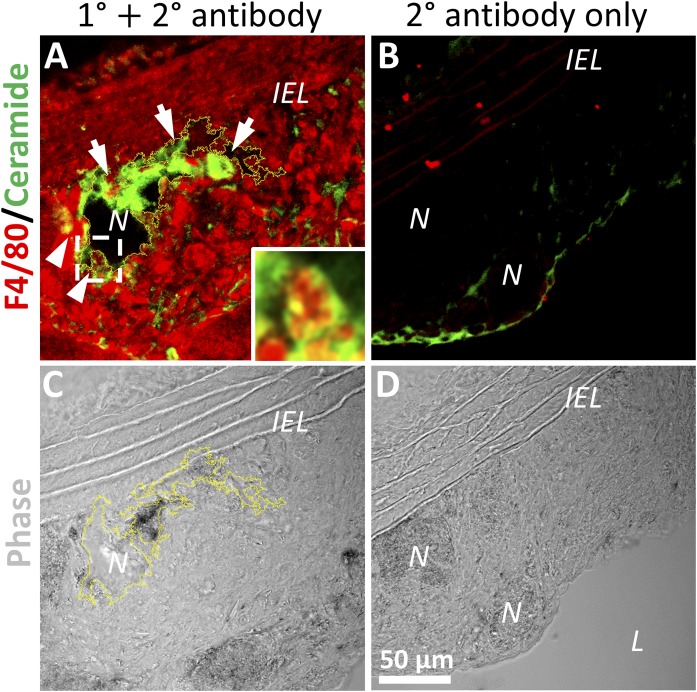
Atherosclerotic lesion macrophages come into contact with and accumulate ceramide. A–D: Aortic sections from hyperlipidemic *ApoE*^−/−^ mice containing atherosclerotic plaque were immunostained for macrophages (F4/80) and ceramide, followed by relevant secondary antibodies (A) or secondary antibodies only (B). Arrows denote macrophages contacting ceramide, and arrowheads show macrophages accumulating ceramide. Inset from dashed box in A shows one such macrophage. Corresponding phase images are also shown (C, D), showing the internal elastic lamina (*IEL*) and necrotic cores (*N*) and lumen (*L*) of the aorta. One necrotic core is outlined in yellow (C).

Lesional LDL becomes enriched in ceramide during atherosclerosis (5). To determine whether macrophages come into contact with such ceramide, we took aortic sections from hyperlipidemic *ApoE*^−/−^ mice and immunostained them for F4/80 to detect macrophages, and for ceramide using a well-characterized anti-ceramide antibody that has been used successfully for staining mouse tissue sections (22). We found that lesional macrophages contact ceramide, particularly in regions close to necrotic cores (**Fig. 1A**, arrows, and Fig. 1C). We also observed macrophages that were accumulating ceramide (Fig. 1A, arrowheads and inset). Sections stained with secondary antibodies alone showed little background staining (Fig. 1B, D). A proportion of ceramide in the plaque is likely to be lesional aggregated LDL, which is known to be enriched in ceramide and accumulate in the subendothelium (intima) of the plaque (5, 23). When macrophages catabolize and internalize this LDL, they accumulate the accompanying ceramide.

### Increasing macrophage ceramide levels inhibits actin polymerization in response to agLDL

**Fig. 2.**
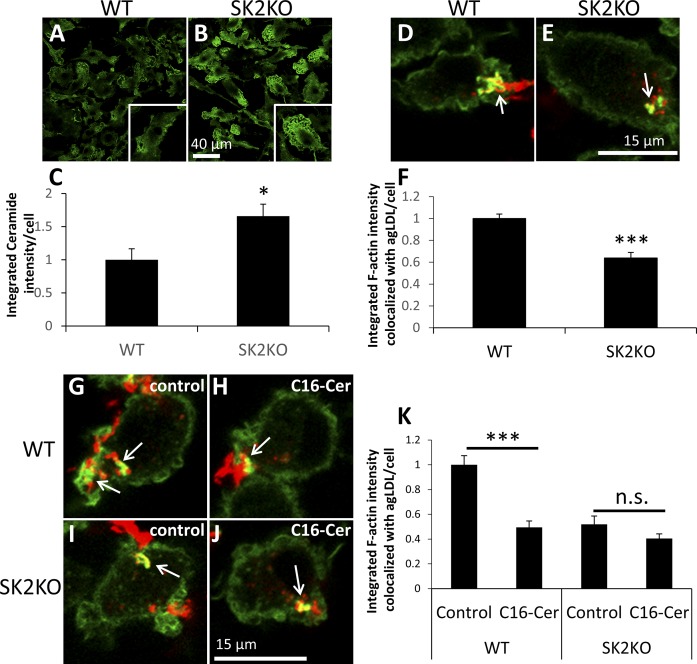
Increasing macrophage ceramide levels inhibits actin polymerization in response to agLDL. A, B: Representative confocal microscopy images of WT and SK2KO macrophages immunostained for ceramide. Individual cells are shown (insets). C: Confocal images were used to quantify ceramide fluorescence per cell. D, E: WT and SK2KO macrophages were treated with Alexa546-agLDL for 1 h prior to fixation and F-actin labeling with Alexa488-phalloidin. Arrows denote macrophage F-actin associated with agLDL. F: Confocal images were used to quantify F-actin colocalized with agLDL for WT and SK2KO macrophages per cell for at least 10 fields containing >100 cells. G–J: WT and SK2KO macrophages were pretreated with methanol (control) (G, I) or 200 μM C16-ceramide in methanol (H, J) for 3 h prior to Alexa546-agLDL treatment for 1 h in the presence of control or C16-ceramide. Cells were fixed and stained for F-actin using Alexa488-phalloidin. Arrows denote macrophage F-actin associated with agLDL. K: Confocal images were used to quantify F-actin colocalized with agLDL for WT and SK2KO macrophages per cell for at least 10 fields containing >100 cells. *P < 0.05; ***P < 0.001, Student’s *t* test. n.s., not statistically significant. Error bars, SEM.

SK2KO macrophages contain increased levels of ceramide (16). Consistent with this, we found that SK2KO BMMs stained more intensely for ceramide than did WT BMMs (**Fig. 2A, B**). Quantification of this showed a 65% increase in ceramide staining in SK2KO compared with WT BMMs (Fig. 2C). We have used SK2KO macrophages to investigate the role of ceramide in the response of BMMs to agLDL. As is discussed below, we found that other changes in the SK2KO BMMs such as decreased S1P or SK1 upregulation cannot explain differences in the response of SK2KO BMMs to agLDL.

We treated WT and SK2KO macrophages with agLDL to assess the effect of SK2 KO on actin polymerization. WT macrophages treated with agLDL displayed robust actin polymerization at the LS (Fig. 2D, arrow), but SK2KO BMMs displayed a 40% reduction in actin polymerization in response to agLDL at the LS (Fig. 2E, arrow, and Fig. 2F). By taking a microscopy approach, we were able to pinpoint changes in actin polymerization in SK2KO BMM, specifically the LS, indicating that these are local and not global phenomena.

SK1 has previously been observed to be significantly upregulated in SK2KO BMMs (16). To assess the role of upregulated SK1 expression in impairment of actin polymerization in response to agLDL observed in SK2KO BMMs, we cultured BMMs deficient in both SK1 and SK2 and analyzed actin polymerization in response to agLDL. SK1 and SK2KO BMMs displayed a similar level of reduction in actin polymerization as did SK2KO BMMs specifically at the LS, suggesting that deficiency of SK2 underlies impaired actin polymerization (supplemental Fig. S1A, arrows, S1B).

The primary function of sphingosine kinases is to phosphorylate sphingosine to generate S1P. S1P can bind a family of S1P receptors (S1P1-5) and can mediate actin reorganization through binding of S1P to receptors S1P1 and S1P2 (24–26). Although it seems unlikely that sufficient S1P would be generated within 60 min of agLDL treatment to accumulate in the culture media, bind to S1P1 or S1P2, and mediate actin polymerization, we nevertheless tested whether lack of S1P production underlies the SK2KO phenotype. We did not observe a reduction in actin polymerization in response to agLDL in either S1P1 KO or S1P2 KO BMMs in comparison with WT (supplemental Fig. S1C–H). This suggests that lack of S1P production and signaling via the S1P receptors cannot explain the SK2KO phenotype.

SK2KO BMMs accumulate both sphingosine and ceramides (16). To determine a specific role for ceramides in reduction of actin polymerization in SK2KO BMMs, we pretreated both WT and SK2KO macrophages with naturally occurring C16-ceramide or solvent control, prior to agLDL treatment. Pretreatment with C16-ceramide led to a 60% reduction in F-actin in WT BMMs versus solvent-treated WT macrophages (Fig. 2G, H, arrows, ). Similar to the phenotype of SK2KO BMM, reduction in actin polymerization was observed only locally at the LS. Pretreatment of SK2KO macrophages with C16-ceramide did not significantly impair actin polymerization in comparison with control solvent-treated SK2KO macrophages (Fig. 2I, J, arrows, ). This shows that increased ceramide impairs actin polymerization locally and specifically at the LS, and no additional effect is seen when ceramide is added to SK2KO macrophages. These data are consistent with impairment of actin polymerization in SK2KO macrophages being due to increased levels of ceramide. To confirm this, we also used C2-ceramide, which can enter cells more easily, and found similar results to those obtained with C16-ceramide (supplemental Fig. S2A–E).

### SK2KO macrophages display impaired foam cell formation in response to agLDL

**Fig. 3.**
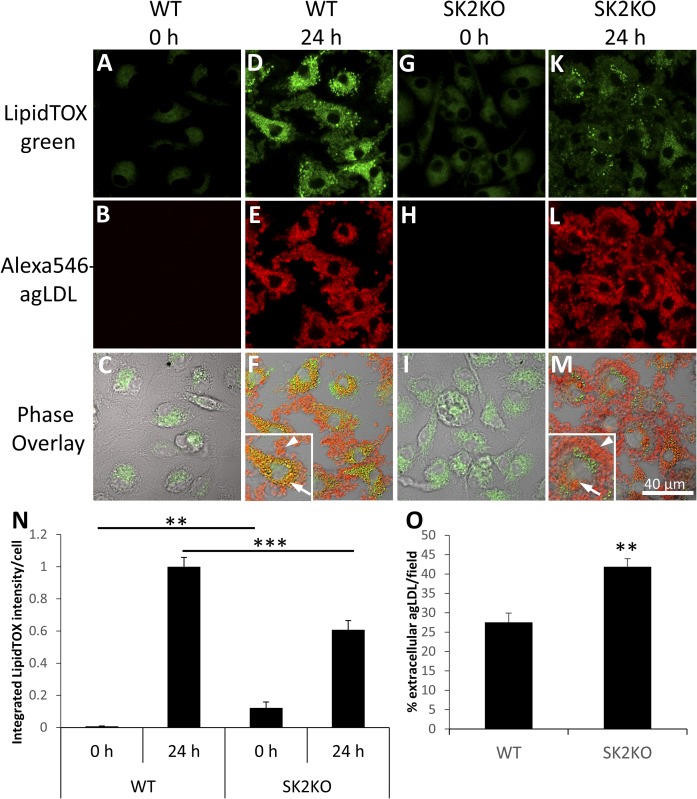
SK2KO macrophages display impaired foam cell formation in response to agLDL. A–M: WT (A–F) and SK2KO (G–M) macrophages were left untreated (A–C, G–I) or treated with Alexa546-agLDL (D–F, K–M) for 24 h prior to fixation and LipidTOX Green staining (A, D, G, K) of neutral lipids. Phase overlay images (C, F, I, M) are also shown. Arrows show internalized Alexa546-agLDL, and arrowheads shows extracellular Alexa546-agLDL. Confocal images were used to quantify neutral lipid content per cell by LipidTOX staining (N) and extracellular agLDL per field (O) for at least 10 fields containing >100 cells. **P < 0.01; ***P < 0.001, one-way ANOVA followed by Bonferroni correction (N) or Student’s *t* test (O). Error bars, SEM.

We have shown previously that actin polymerization at the LS is necessary for agLDL catabolism and foam cell formation (3). We therefore hypothesized that reduced actin polymerization in SK2KO macrophages may lead to impairment of lipid accumulation and foam cell formation. We treated macrophages with agLDL and assessed neutral lipid content. Untreated WT and SK2KO macrophages both displayed low levels of lipid accumulation (**Fig. 3A–C, G–I**). After 24 h treatment with agLDL, SK2KO macrophages displayed a 40% reduction in comparison with WT macrophages in neutral lipid accumulation as assessed by LipidTOX staining (Fig. 3D–F, K–N). Consistent with this, more agLDL had been internalized by WT macrophages (Fig. 3F, inset, arrow) than by SK2KO macrophages (Fig. 3M, inset, arrow), where more agLDL remained extracellular (Fig. 3M, inset, arrowhead). This increase in agLDL that remained extracellular in SK2KO macrophages was confirmed by quantification (Fig. 3O). These results suggest that agLDL catabolism, internalization, and foam cell formation are impaired in SK2KO macrophages when exposed to agLDL.

### Endocytosis of monomeric acLDL and native LDL is not inhibited in SK2KO macrophages

Levels of actin polymerization can strongly influence other mechanisms of LDL uptake such as endocytosis (27). Because we used large aggregates of LDL in this study, we observed little direct endocytosis of agLDL. The main pathway used by macrophages to uptake agLDL is using an extracellular compartment, the LS (27). However, to rule out the possibility of defective endocytosis being the cause of inhibited agLDL catabolism and foam cell formation in SK2KO macrophages, we assessed uptake of monomeric acLDL and native LDL. WT and SK2KO BMMs endocytosed similar amounts of acLDL over 15 min (supplemental Fig. S3A–E). An increase in native LDL endocytosis after 15 min was observed in SK2KO BMMs in comparison with WT (supplemental Fig. S3F–J). Furthermore, staining of F-actin revealed that actin polymerization was still able to occur in SK2KO BMMs at sites of endocytosis in response to both acLDL and native LDL (supplemental Fig. S3D, I, insets). These data rule out a role for endocytosis in impaired agLDL catabolism and foam cell formation observed in SK2KO BMMs.

### SK2KO macrophages display altered morphology

**Fig. 4.**
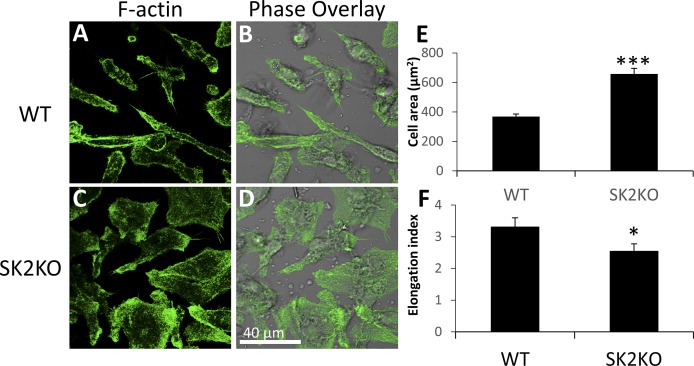
SK2KO macrophages display altered morphology. A–D: Representative confocal microscopy images of F-actin stained with Alexa488-phalloidin in resting WT (A, B) and SK2KO (C, D) macrophages. Phase overlay is also shown. E: Cell area of WT (n = 43) and SK2KO (n = 47) macrophages. F: Elongation indices of WT (n = 40) and SK2KO (n = 40) macrophages. Elongation index = ratio of maximum cell length to maximum cell width. *P < 0.05; ***P < 0.001, Student’ s *t* test. Error bars, SEM.

We observed differences in WT and SK2KO macrophage morphology, which suggested that there were underlying cytoskeletal differences (28). To further characterize the morphology of WT and SK2KO BMMs, we stained cells for F-actin and imaged them in the plane where the cell was attached to the coverslip. This is in contrast to images of BMMs interacting with agLDL (Fig. 1C, D), because agLDL is placed on top of cells, so the confocal slices from the upper half of the cell are shown for those experiments. WT macrophages appeared elongated at the contact point with the coverslip (**Fig. 4A, B**), whereas SK2KO macrophages spread over a larger area (Fig. 4C, D). SK2KO macrophages displayed an 80% increase in cell area in comparison with WT macrophages (Fig. 4E). SK2KO macrophages appeared to lack a bipolar morphology and often displayed a multipolar stellate morphology or an unpolarized circular phenotype. We calculated the elongation index (maximum length/maximum width), and SK2KO macrophages displayed a significantly reduced elongation index in comparison with WT macrophages (Fig. 4F), suggesting that not only the area, but also the shape of cells was altered. These results suggest that under resting conditions, SK2KO macrophages display a morphology distinct from WT macrophages.

### Reduction of RhoA expression restores SK2KO macrophage actin polymerization in response to agLDL

**Fig. 5.**
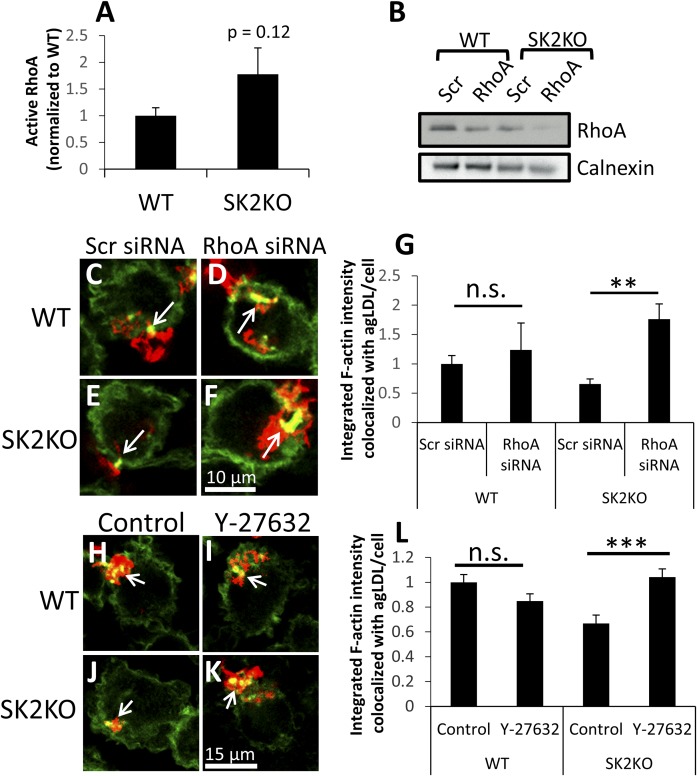
Inhibition of RhoA can restore defective actin polymerization in SK2KO and C16-ceramide-loaded macrophages. A: RhoA activation in resting WT and SK2KO BMMs was assessed using acolorimetric ELISA-based assay. B: WT and SK2KO BMMs were transfected with 2 μM scrambled (Scr) control siRNA or a pool of four different siRNAs targeting RhoA. Seventy-two hours after transfection, protein expression was assessed by immunoblot. Calnex in was used as a loading control. C–F: Scr or RhoA siRNA-transfected cells were treated with Alexa546-agLDL for 1 h prior to fixation and F-actin labeling with Alexa488-phalloidin. Arrows denote macrophage F-actin associated with agLDL. G: Confocal images were used to quantify F-actin colocalized with agLDL for WT and SK2KO macrophages transfected with Scr or RhoA siRNA per cell for at least 10 fields containing >100 cells. H–K: WT and SK2KO macrophages were pretreated with water (control) (H, J) or 20 μM Y-27632 (I, K) for 1 h prior to Alexa546-agLDL treatment for 1 h in the presence of inhibitor. Cells were fixed and stained for F-actin using Alexa488-phalloidin. Arrows denote macrophage F-actin associated with agLDL. L: Confocal images were used to quantify F-actin colocalized with agLDL per cell for at least 10 fields containing >100 cells. **P < 0.01; ***P < 0.001, Student’s *t* test. n.s. not statistically significant. Error bars, SEM.

SK2KO macrophage morphology is similar to that observed previously in *Bcl6^−/−^* macrophages, which was attributed to hyperactivation of RhoA (29). RhoA is an important mediator of the actin cytoskeleton (30). Activation of RhoA increases actin-myosin contraction and can antagonize Rac/Cdc42-dependent actin polymerization (31). We hypothesized that increased RhoA signaling might be responsible for impairment of actin polymerization at the LS in SK2KO macrophages. We therefore measured active RhoA in resting WT and SK2KO macrophages, using an ELISA-based absorbance assay. We observed a trend toward increased RhoA activation in SK2KO macrophages compared with WT; however, this did not quite reach statistical significance (**Fig. 5A**).

To specifically test whether RhoA is responsible for actin polymerization defects in SK2KO BMMs, WT and SK2KO BMMs were transfected with scrambled control siRNA or RhoA- specific siRNA to reduce RhoA protein levels. Seventy-two hours after transfection, immunoblot analysis confirmed that RhoA protein was reduced by RhoA-specific siRNA compared with scrambled siRNA in both WT and SK2KO macrophages (Fig. 5B). SiRNA-transfected cells were then assessed for their ability to polymerize actin in response to agLDL. Although there were no significant differences between control and RhoA siRNA-treated WT macrophages (Fig. 5C, D, arrows), in SK2KO macrophages, RhoA siRNA treatment could significantly rescue actin polymerization specifically at the LS in response to agLDL (Fig. 5E, F, arrows, and ). These results suggest that activation of RhoA in SK2KO macrophages underlies locally impaired actin polymerization at the LS in response to agLDL. In support of these data, locally impaired actin polymerization in response to agLDL caused by C16-ceramide loading of J774 macrophages could be rescued by inhibition of RhoA by Rho inhibitor I (cell-permeable C3 transferase) treatment (supplemental Fig. S4A–E). Efficacy of Rho inhibitor treatment was indicated by the dendritic morphology of Rho inhibitor I-treated WT and SK2KO BMMs observed in the same experiments, known to be induced by Rho inhibition (32) (supplemental Fig. S4F, H, and S4G, I, arrows).

### Inhibition of Rho kinase restores SK2KO macrophage actin polymerization to wild-type levels

RhoA is known to function with Rho kinase for many of its downstream effector processes. To test whether RhoA was working with Rho kinase to antagonize local actin polymerization at the LS in SK2KO macrophages, we pretreated macrophages with a Rho kinase inhibitor (Y-27632) prior to agLDL treatment. Rho kinase inhibition had no significant effect on F-actin at the LS observed in WT macrophages at the LS (Fig. 5H, I, arrows). By contrast, Y-27632 treatment restored local actin polymerization at the LS in SK2KO macrophages to WT levels (Fig. 5J, K, arrows, and ). Phosphorylation of myosin light chains is known to be regulated by Rho kinase (33). Treatment of both WT and SK2KO BMMs with Y-27632 leads to a significant decrease in levels of phospho-MLC2 (supplemental Fig. S5A–E). Therefore, this result confirms the efficacy of Rho kinase inhibition using Y-27632. Changes in phospho-MLC2 might be expected to be observed in SK2KO macrophages that have increased RhoA activity. Compensatory mechanisms are known to exist to retain levels of phospho-MLC2 upon changes in RhoA expression/activity (34) and could occur through changes in activity of myosin light chain kinase or myotonic dystrophy kinase-related Cdc42-binding kinase (35). Together, these data suggest that increased RhoA signaling through Rho kinase is responsible for ceramide-induced impairment of actin polymerization at the LS in SK2KO macrophages.

### RhoA activation can impair actin polymerization and foam cell formation in response to agLDL

**Fig. 6.**
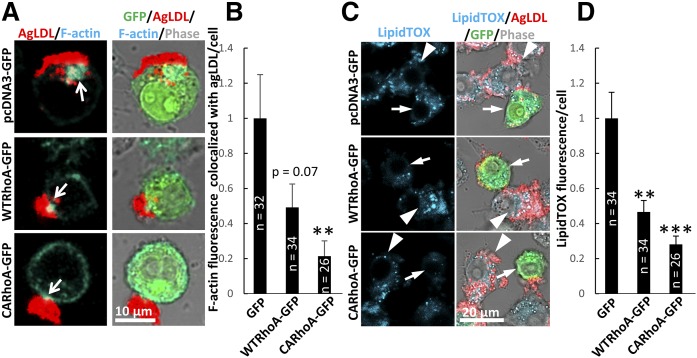
Activation of RhoA is sufficient to impair actin polymerization and agLDL catabolism. A: RAW264.7 macrophages were transiently transfected with pcDNA3-GFP(GFP), pcDNA3-WT RhoA-GFP (WTRhoA-GFP), or pcDNA3-CARhoA-GFP (CARhoA-GFP). Twenty-four hours after transfection, cells were treated with Alexa546-agLDL for 1 h, fixed, and F-actin-labeled with Alexa647-phalloidin. Arrows denote macrophage F-actin associated with agLDL. B: Confocal images were used to quantify F-actin colocalized with agLDL in GFP-transfected cells for given numbers of cells (*n*). C: RAW 264.7 macrophages were transfected with the same plasmids as in A but treated for 6 h with Alexa546-agLDL, fixed, and stained for neutral lipids using LipidTOX Deep Red. Arrows show LipidTOX staining in transfected cells, and arrowheads show LipidTOX staining in nontransfected cells in the same field. D: Confocal images were used to quantify neutral lipid content in GFP-transfected cells for given numbers of cells (*n*). **P < 0.01; ***P < 0. 001, one-way ANOVA followed by Bonferroni correction. Error bars, SEM.

Although reduction of RhoA and inhibition of Rho kinase modulated actin polymerization in SK2KO BMMs, they had a limited effect in WT BMMs (Fig. 5). This raised the question of whether RhoA only modulates actin poly­merization in the presence of excess ceramide (e.g., in SK2KO BMMs). To test this, we assessed actin polymerization in response to agLDL in RAW macrophages transfected with plasmids expressing GFP, wild-type RhoA-GFP (WTRhoA-GFP), or constitutively active (Q63L) RhoA-GFP (CARhoA-GFP). GFP control-expressing cells polymerized actin robustly in response to agLDL (**Fig. 6A**, arrows, and ). WTRhoA-GFP-expressing cells displayed reduced actin polymerization, though this was not quite statistically significant, but CARhoA-GFP-expressing cells displayed significantly reduced actin polymerization specifically at the LS in response to agLDL (Fig. 6A, arrows, and ). These results suggest that global activation of RhoA can inhibit actin polymerization locally at the LS in response to agLDL, even in the absence of excess ceramide.

Actin polymerization promotes agLDL catabolism and foam cell formation, so we tested whether activation of RhoA could impair foam cell formation in response to agLDL. After 6 h agLDL treatment, GFP control-expressing cells were able to accumulate lipid effectively (Fig. 6C, D), whereas expression of either WTRhoA-GFP or CA-RhoA-GFP significantly inhibited lipid accumulation (Fig. 6C, D). These results show that activation of RhoA independently of ceramide can impair actin polymerization and foam cell formation in response to agLDL.

### During agLDL catabolism, ceramide is transferred to macrophages and suppresses actin polymerization

**Fig. 7.**
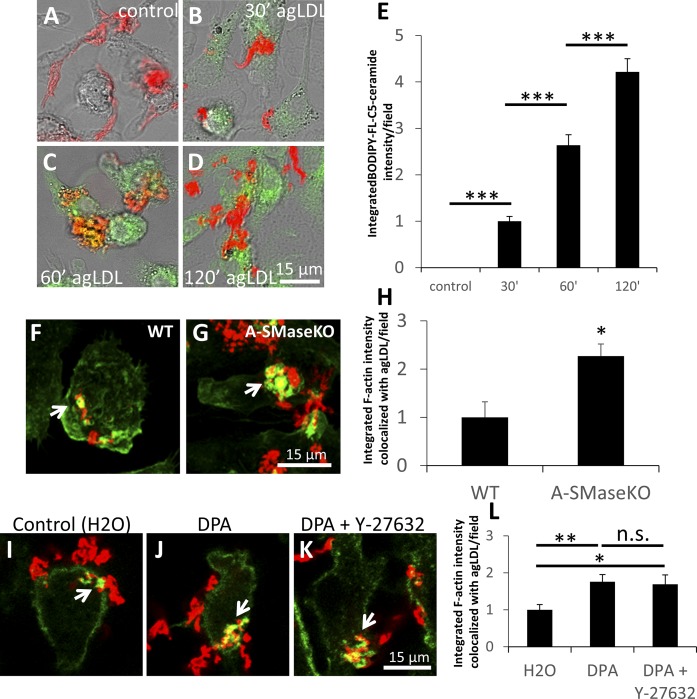
During agLDL catabolism, ceramide is generated by acid SMase, can be transferred to the macrophage, and suppresses actin polymerization in a Rho kinase-dependent manner. A–D: WT macrophages were treated with Alexa546-agLDL for 120 min (A) or Alexa-546-agLDL that had BODIPY-FL-C5-ceramide incorporated into it for 30 (B), 60 (C), or 120 min (D). E: Confocal images were used to quantify cell-associated BODIPY-FL-C5-ceramide for at least 10 fields containing >100 cells. F, G: WT or A-SMaseKO macrophages were treated with Alexa546-agLDL for 1 h prior to fixation and F-actin labeling with Alexa488-phalloidin. Arrows denote macrophage F-actin associated with agLDL. H: Confocal images were used to quantify F-actin colocalized with agLDL for WT and A-SMaseKO macrophages per cell for at least 10 fields containing>100 cells. I–K: WT macrophages were pretreated with control (H_2_O) (I), 400 nM desipramine (DPA) (J), and 400 nM DPA + 20 μM Y-27632 (K) for 30 min prior to treatment with Alexa546-agLDL for 1 h in the presence of inhibitors. Cells were fixed and stained for F-actin using Alexa488-phalloidin. Arrows denote macrophage F-actin associated with agLDL. L: Confocal images were used to quantify F-actin colocalized with agLDL in control or inhibitor-treated J774 macrophages for at least 10 fields containing >100 cells. *P < 0.05; **P < 0.01; ***P < 0.001, one-way ANOVA followed by Bonferroni correction. n.s. not statistically significant. Error bars, SEM.

We have observed previously that lysosome exocytosis occurs at the LS to aid agLDL catabolism (2). Lysosomes contain L-SMase, which functions more effectively at an acidic pH (which occurs at the LS). Therefore, we wondered whether generation of ceramide in the LS during agLDL catabolism might modulate actin polymerization at the LS. For this to occur, ceramide from agLDL would have to be capable of being transferred to the macrophage. To test this, we incorporated BODIPY-FL-C5-ceramide into LDL prior to aggregation and incubated the labeled agLDL with wild-type macrophages. Macrophages treated with control agLDL with no incorporation of BODIPY-FL-C5-ceramide showed no fluorescence, as was expected (**Fig. 7A**), whereas in macrophages treated with BODIPY-FL-C5-ceramide in the agLDL, internalization of BODIPY-FL-C5-ceramide was observed as early as 30 min (Fig. 7B–E). This shows that ceramide can enter the cell during agLDL catabolism.

A likely source of ceramide at the LS is generated by L-SMase (delivered by lysosome exocytosis) and cleavage of sphingomyelin in agLDL. To test this, we assessed actin polymerization at the LS in macrophages from WT or A-SMase KO mice (deficient in acid SMase, both L-SMase and S-SMase). A-SMaseKO macrophages displayed a 2.2-fold increase in actin polymerization specifically at the LS over WT macrophages (Fig. 7F, G, arrows, and ). These results confirmed a role for A-SMase and ceramide in actin polymerization at the LS. To further investigate this result, and to specifically test the role of L-SMase in this process, we incubated wild-type macrophages with control (H_2_O) or an L-SMase-specific inhibitor desipramine (DPA) to prevent ceramide generation at the LS from agLDL catabolism. DPA is a cationic amphiphile that accumulates in the acidic compartment and induces proteolysis of mature L-SMase (36, 37). Inhibition of L-SMase using DPA displayed a 1.7-fold increase in actin polymerization at the LS (Fig. 7I, J, arrows, and ). Simultaneous inhibition of both L-SMase and Rho kinase using DPA and Y-27632 treatment did not significantly increase actin polymerization above levels observed for DPA alone (Fig. 7K, arrows, and ). These results suggest that if ceramide is not generated during agLDL catabolism, RhoA signaling is not activated, and actin polymerization at the LS is enhanced. These data are consistent with a role for ceramide in the inhibition of actin polymerization at the LS during agLDL catabolism through a RhoA/Rho kinase-dependent mechanism.

## DISCUSSION

A key event in the initiation of atherosclerosis is the retention of LDL in the subintima (1). This LDL is aggregated and avidly bound to the extracellular matrix, so it cannot be internalized by standard endocytic mechanisms. Macrophages use a specialized compartment, the LS, aided by local actin polymerization, to partially digest this LDL prior to internalization. This leads to foam cell formation, a process central to the pathogenesis of atherosclerosis. Foam cells are vulnerable to cell death, which releases cellular cholesterol into the surrounding area, exacerbating atherosclerosis. Therefore, identification of regulatory mechanisms that can limit the extent of foam cell formation is of importance. Herein, we describe a RhoA/Rho kinase-dependent regulatory pathway, activated by ceramide, which suppresses actin polymerization and limits the extent of agLDL catabolism and foam cell formation in response to agLDL. We also show that the process of agLDL catabolism can cause ceramide generation from agLDL, and this can further inhibit actin polymerization through RhoA/Rho kinase. This mechanism is likely to play a role in vivo as ceramide is enriched in the plaque microenvironment and in lesional LDL (5).

Previous studies have highlighted a role for ceramide in the activation of RhoA in other cell types. Treatment of endothelial cells with ceramide was able to activate RhoA, though this may be due to metabolism of ceramide to sphingosine and then S1P (38). A more recent study found that microbial neutral SMase treatment of epithelial cells increases ceramide and activates RhoA (39). Another study found double the amount of RhoA activity in renal mesangial cells from SK2KO mice compared with WT (40). In fibroblasts, generation of ceramide stimulates sphingosine kinase activity downstream of PI 3-kinase to activate RhoA (30). In intestinal epithelial cells, generation of ceramide through microbial SMase can activate RhoA more directly and can protect against *Salmonella* invasion through reorganization of the F-actin network (39).

In the context of atherosclerosis, secretion of S-SMase has been shown to catabolize LDL (as LDL contains sphingomyelin), but this catabolism is partial and leads to fusion of LDL particles to form agLDL (41, 42). This SMase-induced aggregation promotes lipoprotein retention in arterial walls, and KO of acid SMase in hyperlipidemic mice reduced early aortic root lesion area by 40%–50% (42). Furthermore, LDL has been shown to modulate acid SMase activity. Oxidized LDL contained in immune complexes have been shown to activate macrophage L-SMase and S-SMase activity, and disruption of L-SMase activity can inhibit internalization of these complexes (43). Although ceramide can play a detrimental role in many settings, recent studies have emphasized several diverse functions of ceramide are as a second messenger. One such example of this transpires during plasma membrane repair. Lysosome exocytosis stimulated by damage to the plasma membrane can deliver L-SMase to the cell surface, where sphingomyelin in the membrane is cleaved into ceramide to generate ceramide-rich membrane patches (44). These patches denote lesions that contain damaged membrane, and they are rapidly internalized by the cell and targeted for degradation (44). Therefore, ceramide can play a protective role within the cell under some circumstances.

In this study, we show data suggesting that ceramide accumulated within the cell can inhibit actin polymerization specifically at the LS (Fig. 2) and that global activation or inactivation of RhoA/Rho kinase is sufficient to modulate actin polymerization at the LS (Figs. 5, ). Ceramide is also generated by agLDL catabolism at the LS (Fig. 7). Such ceramide can inhibit local actin polymerization at the LS, through a RhoA/Rho kinase-dependent mechanism. Ceramide generated at the LS may redistribute quickly, and others have shown that ceramide has an estimated lateral diffusion coefficient of 1 μm/s in a liquid-crystalline phase (45). Therefore, though we were able to show that global changes in ceramide/RhoA/Rho kinase could modulate actin polymerization in specific domains (at the LS), we were unable to determine whether ceramide generated at the LS could act locally to modulate the process. Such changes occurring in specific domains will be significant for macrophage biology, and the relationship between agLDL catabolism and other critical processes, such as endocytosis [known to occur from ceramide-rich domains (44)], cell adhesion to extracellular matrix, and cell migration (local activation of RhoA is known to induce inactivation of Rac/Cdc42 at those sites) (46). Further studies should focus on the importance of such local changes at the LS.

This study has characterized the effect of ceramide on inhibition of agLDL catabolism and foam cell formation, which may be considered a potentially beneficial effect on atherosclerosis. Therefore, increasing RhoA activation may represent a novel pathway that may be exploited therapeutically, and increasing macrophage-specific activation of RhoA was shown to reduce atherosclerosis in mice (47). As was discussed previously, ceramide is a critical mediator of cell apoptosis. The proximity of ceramide-loaded macrophages to necrotic cores in atherosclerotic lesions is particularly striking (Fig. 1A). This raises the possibility that ceramide might be mediating cell death in atherosclerotic lesions, a process that exacerbates atherosclerosis. Increased RhoA activation has been observed in advanced versus early atherosclerotic lesions in hyperlipidemic rabbits (48), and RhoA activity is known to impair Rac/Cdc42 activation (31). Therefore, activation of RhoA by ceramide in atherosclerotic lesions might impair macrophage motility and emigration from the lesion. Macrophage susceptibility to apoptosis and inhibited emigration from plaques are major contributing factors to the pathogenesis of atherosclerosis, so further studies are necessary to dissect the role of Rho-dependent signaling in macrophage biology during atherosclerosis.

## Supplementary Material

Supplemental Data
