# Supplementary material for: Ceramide activation of RhoA/Rho kinase impairs actin polymerization during aggregated LDL catabolism
Source: J Lipid Res. 2017 Aug 16;58(10):1977–87. doi: 10.1194/jlr.M076398 (PMC5625121; doi:10.1194/jlr.M076398)
Supplement: Supplemental Data [file supp_58_10_1977__index.html]

Ceramide Activation of RhoA/Rho Kinase Impairs Actin Polymerization during Aggregated LDL Catabolism — Ceramide activation of RhoA/Rho kinase impairs actin polymerization during aggregated LDL catabolism — Supplemental Data 

# Ceramide activation of RhoA/Rho kinase impairs actin polymerization during aggregated LDL catabolism

## Supplemental Data

- Supplemental Figures S1-S5 (.pdf, 963 KB) - Combined PDF of all supplemental files
