# Supplementary material for: Ceramide activation of RhoA/Rho kinase impairs actin polymerization during aggregated LDL catabolism
Source: J Lipid Res. 2017 Aug 16;58(10):1977–87. doi: 10.1194/jlr.M076398 (PMC5625121; doi:10.1194/jlr.M076398)
Supplement: Supplemental Data [file 10.1194_M076398_jlr.M076398-1.pdf]

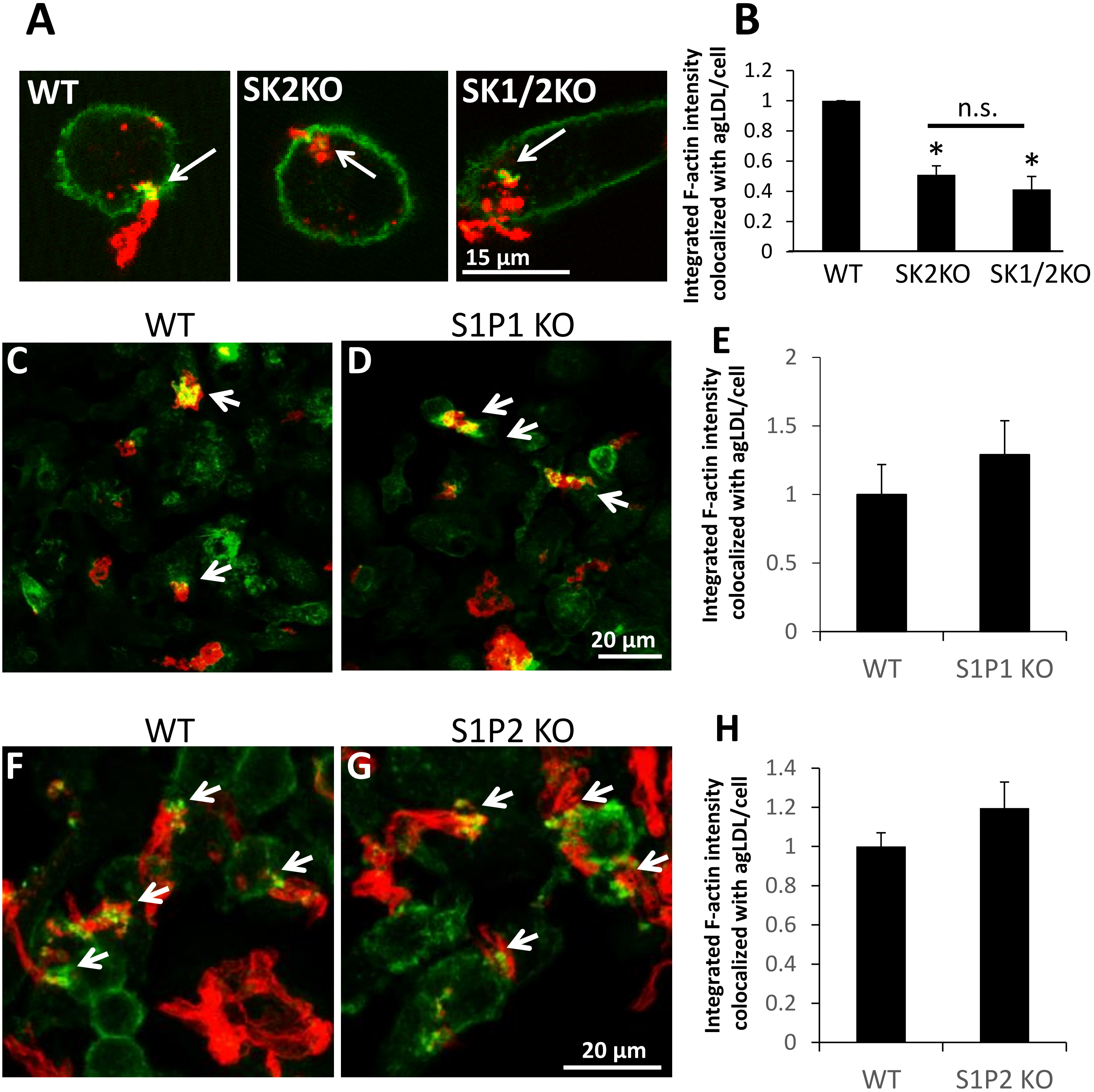

Figure S1. Sphingosine Kinase 2 KO BMMs display impaired actin polymerization in response to agLDL, but S1P1 and S1P2 KO BMMs do not. (A) WT, SK2KO and SK1/2KO macrophages were treated with Alexa546-agLDL (red) for 1 h prior to fixation and F-actin labeling with Alexa488-phalloidin (green). Arrows denote macrophage F-actin associated with agLDL. **(B)** Confocal images were used to quantify F-actin colocalized with agLDL for WT and SK2KO macrophages per cell for at least 10 fields containing >100 cells. **(C-D)** WT and S1P1 KO macrophages were treated with Alexa546-agLDL for 1 h prior to fixation and F-actin labeling with Alexa488-phalloidin. Arrows denote macrophage F-actin associated with agLDL. **(E)** Confocal images were used to quantify F-actin colocalized with agLDL for WT and S1P1 KO macrophages per cell for at least 10 fields containing >100 cells. **(F-G)** WT and S1P2 KO macrophages were treated with Alexa546-agLDL for 1 h prior to fixation and F-actin labeling with Alexa488-phalloidin. Arrows denote macrophage F-actin associated with agLDL. **(H)** Confocal images were used to quantify F-actin colocalized with agLDL for WT and S1P2 KO macrophages per cell for at least 10 fields containing >100 cells. * $p < 0.05$ one-way ANOVA followed by Bonferroni correction. n.s. not statistically significant. Error bars s.e.m.

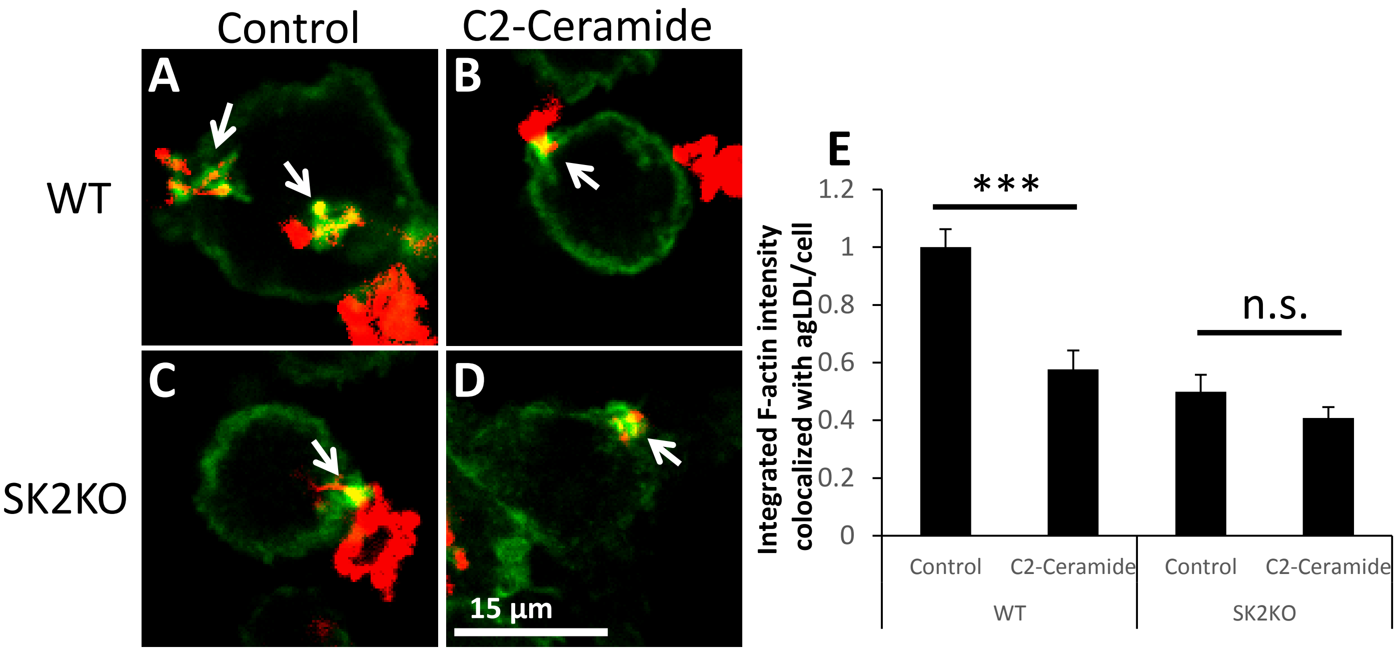

Figure S2. Increasing macrophage ceramide levels inhibits actin polymerization in response to agLDL. (A-D) WT and SK2KO macrophages were pre-treated with MeOH (control) (A, C)) or 10 µM C2-ceramide in methanol (B, D) for 1 h prior to Alexa546-agLDL treatment for 1 h in the presence of control or C2-ceramide. Cells were fixed and stained for F-actin using Alexa488-phalloidin. Images shown are sum projections of z-stacks, and arrows denote macrophage F-actin associated with agLDL. **(E)** Confocal images were used to quantify F-actin colocalized with agLDL for WT and SK2KO macrophages per cell for at least 10 fields containing >100 cells. *** $p < 0.001$ one-way ANOVA followed by Bonferroni correction. n.s. not statistically significant. Error bars s.e.m.

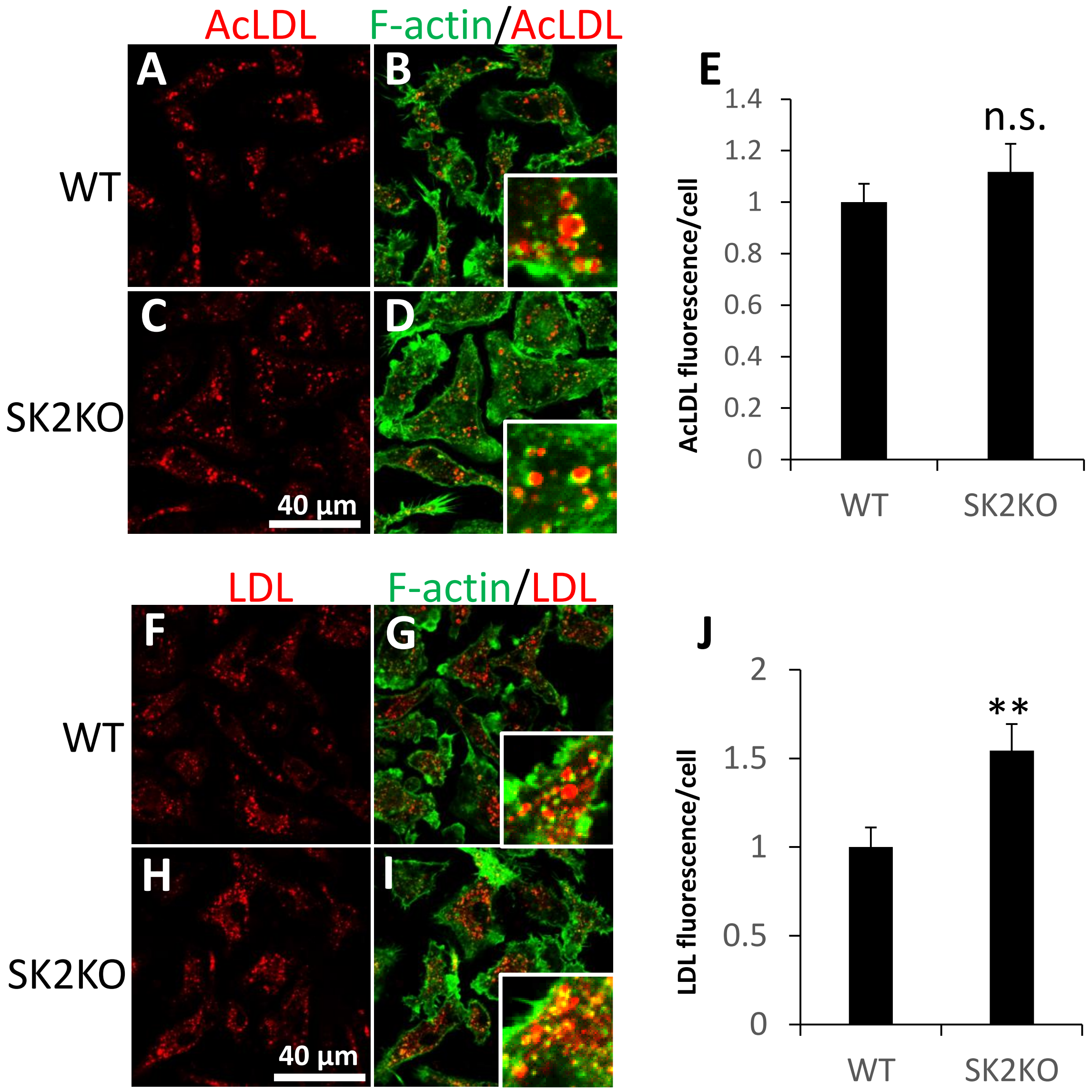

Figure S3. Uptake of monomeric acLDL or native LDL are not impaired in SK2KO BMMs. (A-D) WT and SK2KO macrophages were treated with 50 µg/mL Alexa546-acLDL (A, C) for 15 min, prior to fixation and staining for F-actin using Alexa488-phalloidin (B, D). Insets show F-actin colocalized with acLDL containing endosomes. **(E)** Confocal images were used to quantify acLDL contained in WT and SK2KO macrophages per cell for at least 10 fields containing >100 cells. **(F-I))** WT and SK2KO macrophages were treated with 50 µg/mL Alexa546-LDL (F, H) for 15 min, prior to fixation and staining for F-actin using Alexa488-phalloidin (G, I). Insets show F-actin colocalized with LDL containing endosomes. **(J)** Confocal images were used to quantify LDL contained in WT and SK2KO macrophages per cell for at least 10 fields containing >100 cells. ** $p < 0.001$ student's t test. n.s. not statistically significant. Error bars s.e.m.

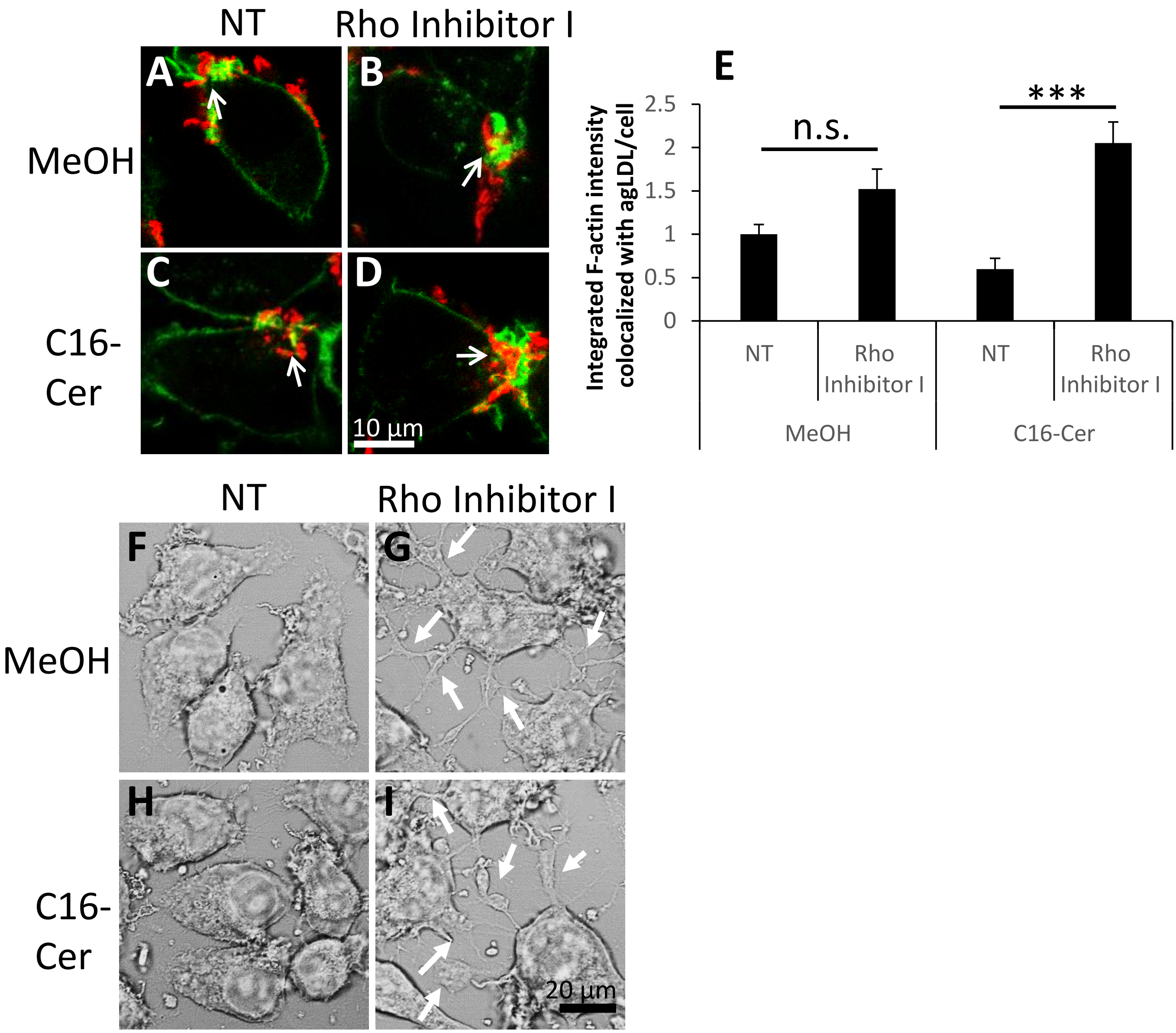

Figure S4. Inhibition of RhoA can restore defective actin polymerization in response to agLDL in C16-ceramide loaded macrophages. (A-D) J774 macrophages were left untreated (NT) (A, C) or pre-treated for 3 h with 2 µg/mL Rho Inhibitor I (B, D) in the presence of MeOH control (A, B) or 200 µM C16-ceramide (C, D) prior to 1 h incubation with Alexa546-agLDL in the presence of the same compounds. Cells were fixed and stained for F-actin using Alexa488-phalloidin. Arrows denote macrophage F-actin associated with agLDL. **(E)** Confocal images were used to quantify F-actin colocalized with agLDL per cell for at least 10 fields containing >100 cells. **(F-I)** Brightfield images of J774 macrophages from A-D. Arrows highlight the dendritic morphology induced by Rho Inhibitor I treatment. *** $p < 0.001$ student's t test. n.s. not statistically significant. Error bars s.e.m.

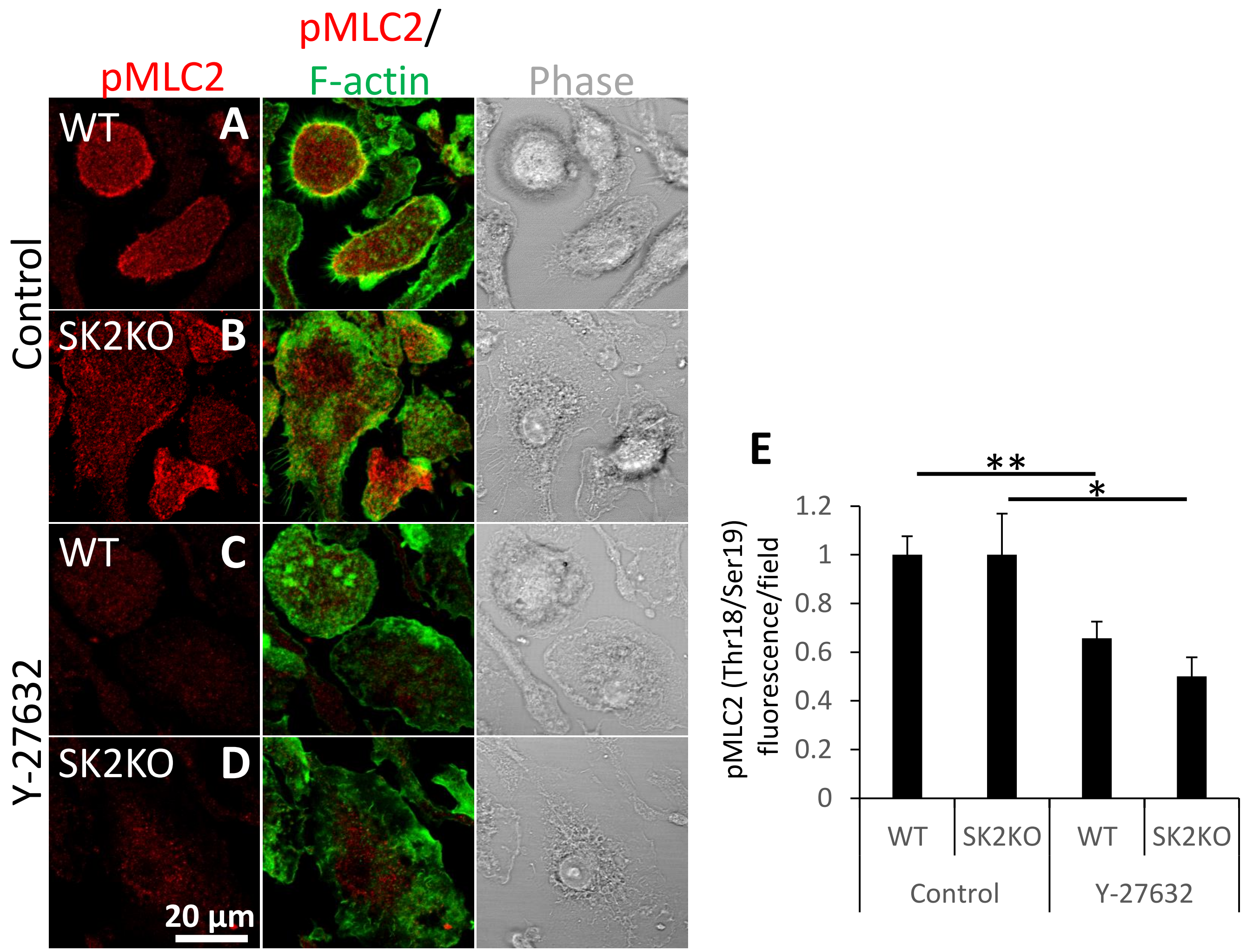

Figure S5. Treatment of WT and SK2KO macrophages with Y-27632 reduces levels of phosphorylated Myosin Light Chain 2. (A-D) WT (A, C) and SK2KO (B, D) macrophages were left untreated (Control) (A, B) or treated for 1 h with 20 μM Y-27632 (C, D). Cells were fixed and stained for phospho-Myosin Light Chain 2, followed by Alexa546 labelled secondary antibody, and F-actin using Alexa488-phalloidin. **(E)** Confocal images were used to quantify phospho-Myosin Light Chain 2 fluorescence for at least 10 fields containing >100 cells. * $p < 0.05$, ** $p < 0.01$ student's t test. n.s. Error bars s.e.m.
